# A Mendelian randomization study of the entire phenome to explore the causal links between epilepsy

**DOI:** 10.1002/brb3.3602

**Published:** 2024-06-19

**Authors:** Wei Zhang, Li‐Ming Zhang, Lin Zhi, Ji Qi, Jue He

**Affiliations:** ^1^ Department of Neurosurgery Beijing Fengtai Hospital Beijing China; ^2^ Department of Neurosurgery Jinan University Affiliated 999 Brain Hospital Guangzhou China

**Keywords:** biomarker, epilepsy, lifestyle, Mendelian randomization, phenome

## Abstract

**Objective:**

The causes and triggering factors of epilepsy are still unknown. The results of genome‐wide association studies can be utilized for a phenome‐wide association study using Mendelian randomization (MR) to identify potential risk factors for epilepsy.

**Methods:**

This study utilizes two‐sample MR analysis to investigate whether 316 phenotypes, including lifestyle, environmental factors, blood biomarker, and more, are causally associated with the occurrence of epilepsy. The primary analysis employed the inverse variance weighted (IVW) model, while complementary MR analysis methods (MR Egger, Wald ratio) were also employed. Sensitivity analyses were also conducted to evaluate heterogeneity and pleiotropy.

**Results:**

There was no evidence of a statistically significant causal association between the examined phenotypes and epilepsy following Bonferroni correction (*p* < 1.58 × 10^−4^) or false discovery rate correction. The results of the MR analysis indicate that the frequency of tiredness or lethargy in the last 2 weeks (*p* = 0.042), blood uridine (*p* = 0.003), blood propionylcarnitine (*p* = 0.041), and free cholesterol (*p* = 0.044) are suggestive causal risks for epilepsy. Lifestyle choices, such as sleep duration and alcohol consumption, as well as biomarkers including steroid hormone levels, hippocampal volume, and amygdala volume were not identified as causal factors for developing epilepsy (*p* > 0.05).

**Conclusions:**

Our study provides additional insights into the underlying causes of epilepsy, which will serve as evidence for the prevention and control of epilepsy. The associations observed in epidemiological studies may be partially attributed to shared biological factors or lifestyle confounders.

## Graphical abstract

1. There was no robust statistically significant casual association between any of the 316 phenotypes and epilepsy. 2. There is a suggestive causal relationship between Frequency of tiredness or lethargy in last 2 weeks, blood free cholesterol, blood uridine, blood propionylcarnitine and epilepsy.

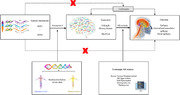

## 1 INTRODUCTION

Globally, epilepsy affects over 70 million individuals, making it one of the most prevalent brain disorders. It is characterized by a persistent tendency to generate spontaneous epileptic seizures, which can result in a range of neurobiological, cognitive, and psychosocial impacts (Fisher et al., 2014; Thijs et al., 2019). Research findings suggest that epilepsy accounts for more than 0.5% of the world's illness, leading to considerable financial repercussions, such as medical costs, premature death, and a decrease in productivity (de Boer et al., 2008; Fazel et al., 2013; Nevalainen et al., 2016). Even though only a portion of fortunate patients can find and remove the epileptic focus by referring to the tips for stereoelectroencephalography to achieve long‐term relief. However, early induction of seizures after electrode implantation is a challenge and uncertain epileptic seizure onset time indirectly leads to an increased treatment burden for epilepsy. Therefore, it is essential to investigate the etiology and precipitating factors of epilepsy.

Exploring the triggers of epilepsy has two potential applications for reducing healthcare costs. One goal is to decrease the frequency of seizures and alleviate the burden of the disease, while the other is to trigger seizures during hospital monitoring to enable prompt and effective monitoring of seizure activity, potentially resulting in reduced hospitalization expenses. Although previous research has found that some factors are associated with the occurrence of epilepsy, such as sleep (Moro et al., 2023), education (Kobau et al., 2023), and blood metabolites (Cai et al., 2022). Most of these studies, however, only establish an association between exposure and epilepsy and do not establish a causal relationship, and even some of the conclusions are conflicting. Vulnerable to reverse causality, confounding bias, and high‐frequency exposure, observational studies such as these can lead to unreliable conclusions about the causal relationship between the disease phenotype and the outcome (Spiller et al., 2019). Exploring the cause of epilepsy, a scientific method is necessary to devise preventive or triggering strategies to lessen its burden, yet it should not be as laborious or time‐consuming as Randomized controlled clinical trial (RCT) studies and should ideally be cost efficient.

Mendelian randomization (MR) is a statistical technique that employs genetic variants as instrumental variables (IVs) to assess the causal effect of exposures on the onset of illness (Burgess et al., 2013; Lawlor et al., 2008). Research has already been conducted to explore the risk factors for epilepsy that have been discussed in traditional epidemiological studies, such as the absence of a causal relationship between drinking coffee and epilepsy, while a causal relationship exists between smoking or alcohol and epilepsy (Larsson & Burgess, 2022; Zhang et al., 2022). In the past, the results of these factors were based on correlation algorithms and empirically interpreted by us as having a correlation. The above‐mentioned research is based on hypotheses regarding the etiology of the disease, aiming to gradually provide answers to problem‐solving for the daily lives of epilepsy patients.

Based on these requirements and objectives, we have employed a strategy that integrates phenome‐wide association study (Hemani et al., 2018) and MR to identify the etiology of epilepsy to confirm documented correlations and explore potential new causal relationships that may have gone unnoticed in prior research. Our analysis involved the investigation of 316 distinct phenotypes, totaling 8387 single‐nucleotide polymorphisms (SNPs) (Saunders et al., 2021), in conjunction with a summary of genetic information obtained from a genome‐wide association studies (GWAS) of epilepsy. The study cohort included 6850 cases and 960,974 control subjects totally (Dönertaş et al., 2021; International League Against Epilepsy Consortium on Complex Epilepsies, 2018; Jiang et al., 2021). Additionally, the factors affecting cancer are included in the scope of the study because some brain tumors are thought to be capable of causing epilepsy (van Opijnen et al., 2023).

## 2 METHODS

### 2.1 Genetic instruments for phenotypes

**FIGURE 1:**
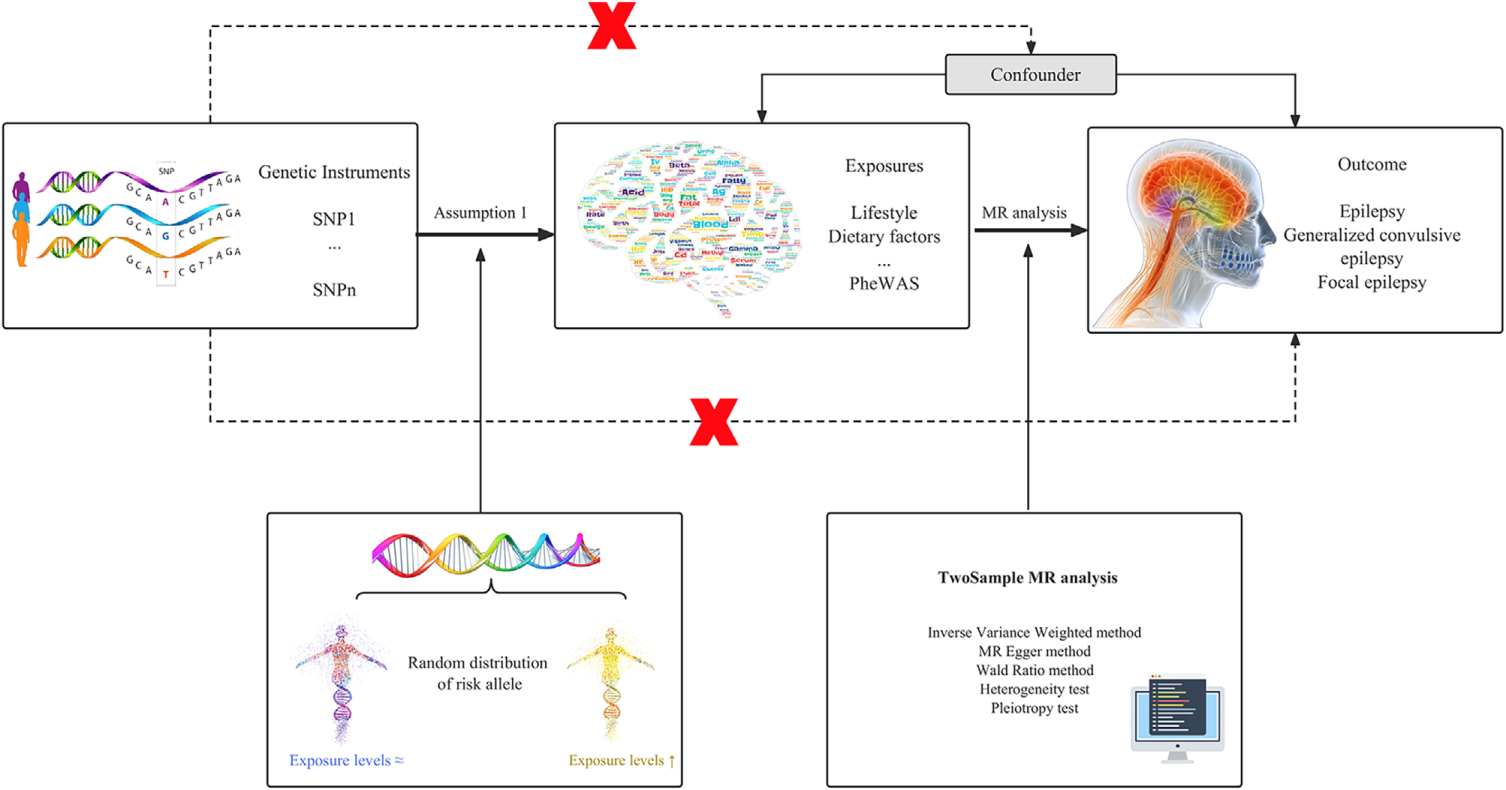
The framework for Mendelian randomization studies involves utilizing genetic variants that are known to be associated with the exposure in question (based on the nonzero effect assumption) to estimate whether the exposure has a causal effect on the outcome of interest. The key assumptions underlying this approach are that the genetic variable is independent of potential confounders (based on the independence assumption) and that it does not directly affect the outcome of interest (based on the exclusion restriction assumption). MR, Mendelian randomization; PheWAS, phenome‐wide association study; SNP, single‐nucleotide polymorphisms.

To test the genetically predicted effects of liability toward 316 phenotypes on epilepsy risk, we used SNPs as genetic instruments. We guarantee that the rsID, effect size (beta), standard error of the effect size (se), and allele of the SNP exhibiting the effect (effect_allele) for each SNP are all acquired. Moreover, to accurately estimate the magnitude of the causal effect, linear relationships and no interactions are essential (Figure 1) (Burgess et al., 2013; Lawlor et al., 2008). The chosen SNPs must meet three specific requirements: (1) they should have a significant association with genetic instrumentation at a genome‐wide significance threshold of *p* < 5 × 10^−8^, (2) they should be screened for linkage disequilibrium interference, with linkage disequilibrium (LD) *r* ^2^ < 0.01 to ensure that there is no correlation between the SNPs and that their pairing is entirely random, and (3) they should not be affected by any other potential risk factors (Cai et al., 2022; Choi et al., 2019; Yang et al., 2020). We exclusively focused on analyzing continuous traits, since binary traits (such as disease status) analyzed with binary outcomes in two‐sample MR frameworks have a higher likelihood of producing inaccurate causal estimates (Disney‐Hogg et al., 2018; Didelez et al., 2010; Palmer et al., 2023).

### 2.2 Epilepsy data

The classification system for epilepsy is complex and can generally be summarized into three types: epilepsy, generalized convulsive epilepsy, and focal epilepsy. In order to understand the causal relationships between IVs and different types of epilepsy, as well as whether the results are robust, we used three datasets that cover these three types. Overall, the GWAS data consisted of 6850 cases, including 3900 individuals with epileptic (Dönertaş et al., 2021), 290 with generalized convulsive epilepsy (Jiang et al., 2021), and 2660 with focal epilepsy (International League Against Epilepsy Consortium on Complex Epilepsies, 2018). The control group comprised a total of 960,974 individuals, including 480,698 cases in the epileptic group, 456,058 cases in the generalized convulsive epilepsy group, and 24,218 cases in the focal epilepsy group. All study participants were of European ancestry. Genetic datasets for three types of epilepsy were sourced from the public GWAS dataset of the International Federation of Industrial Universities Project (https://gwas.mrcieu.ac.uk) (Table S1C).

### 2.3 Mendelian randomization analysis

An evaluation was conducted with the “TwoSampleMR” and “MRInstruments” R packages to carry out a two‐sample MR analysis (Hemani et al., 2018; Rasooly & Peloso, 2021). The SNPs employed as genetic tools to examine the traits were determined through recent meta‐analyses, carefully selected by MR‐Base (Table S1A,B) (Hemani et al., 2018). We obtained the chromosome position, effect size in standard deviations per allele, and corresponding standard error for each SNP. For the ultimate analysis, we concentrated exclusively on SNPs that had the most noteworthy effect on the trait (Table S2A–C). After applying clumping, we computed the *F*‐statistic and *r* ^2^. *F*‐statistic ≥ 10 are considered strong instruments and will be included in the study. In the case of traits with multiple SNPs acting as IVs, a random‐effects inverse‐weighted variance (IVW‐RE) model was mainly utilized to estimate the causal effects, assuming that each SNP represents a distinct causal effect.

We employed a Bonferroni correction to tackle the problem of multiple testing, establishing a significance threshold of *p* ≤ 1.58 × 10^−4^(0.05/316) as statistically significant (Hochberg, 1988). Nevertheless, we recognize that the Bonferroni correction can be overly cautious and may lead to a high rate of false negatives (Perneger, 1998).To address this, we also executed false discovery rate (Benjamini and Hochberg method) correction (Benjamini & Hochberg, 1995) (Table S3A–C). Should the *p*‐value adjusted stay below 0.05, it was deemed statistically significant. If the unadjusted *p*‐value was below 0.05 but the adjusted *p*‐value was above 0.05, it was indicative of a suggestive causal relationship.

To ensure the reliability of our MR analysis, we conducted sensitivity analysis (Table S4A–C). The presence of heterogeneity among estimates was evaluated using the *I* ^2^ statistic and the Cochran *Q* test. Statistical significance was determined by Cochran *Q*­derived *p* < 0.05 and an *I* ^2^ > 50% (Higgins et al., 2003; Higgins & Thompson, 2002) (Table S4A–C). The assessment of directional pleiotropy (Hemani et al., 2018) was conducted using MR‐Egger regression (Bowden et al., 2015) (Table S5A–C). Then, we examined the potential impact of outlying and pleiotropic SNPs on causal estimates using a leave‐one‐out strategy (Table S6)

## 3 RESULTS

### 3.1 Epilepsy

**FIGURE 2:**
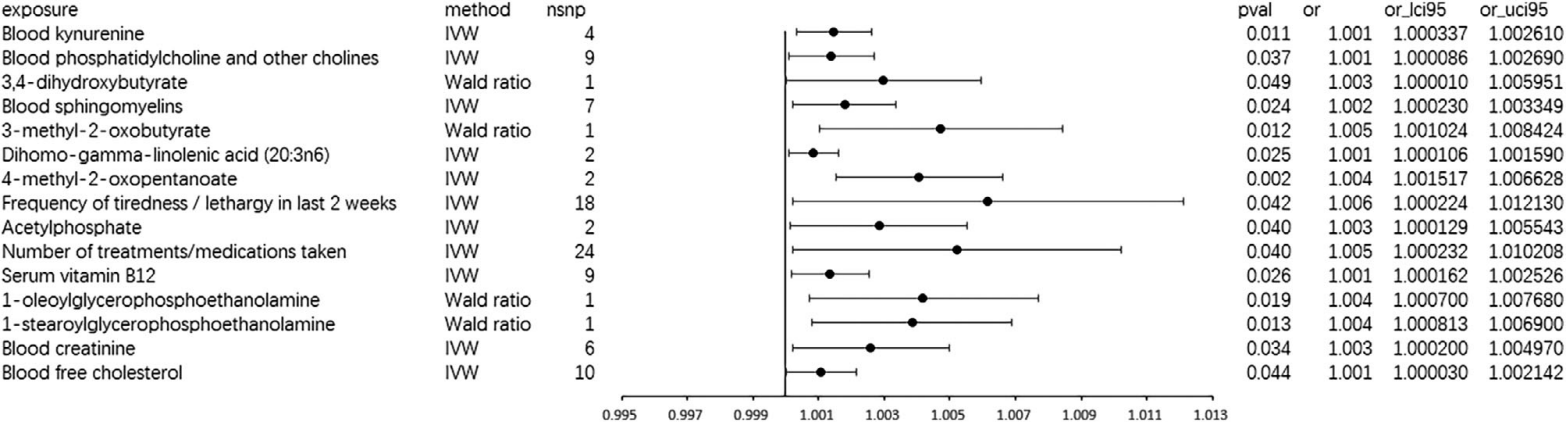
Odds ratios assessing the links between genetically predicted exposure and the occurrence of epilepsy. The estimates correspond to Blood kynurenine, Blood phosphatidylcholine, and other cholines, 3,4‐dihydroxybutyrate, Blood sphingomyelins, 3‐methyl‐2‐oxobutyrate, Dihomo‐gamma‐linolenic acid (20:3n6), 4‐methyl‐2‐oxopentanoate, Frequency of tiredness / lethargy in last 2 weeks, Acetylphosphate, Number of treatments/medications taken, Serum vitamin B12, 1‐oleoylglycerophosphoethanolamine, 1‐stearoylglycerophosphoethanolamine, Blood creatinine, and Blood free cholesterol.

From a genetic perspective, there is suggestive evidence of a causal relationship between the frequency of tiredness or lethargy in the last 2 weeks and an increased risk of developing epilepsy (Figure 2) with an odds ratio (OR) for this association of 1.006 (95% CI 1.0002–1.0121; *p* = 0.042) under IVW model. The Cochran *Q* test and *I* ^2^ indicated no significant heterogeneity (Cochran *Q*­derived *p* = 0.354; *I* ^2^ = 8.444; Table S4A), and there was no evidence for unbalanced pleiotropy (MR­Egger intercept, *p* = 0.791; Table S5A). However, other factors that represent physical exertion did not suggest an increased risk of epilepsy through genetic causes. For example, job involving heavy manual or physical work (beta = −0.005, se = 0.002, *p* = 0.04), job involving mainly walking or standing (beta = −0.008, se = 0.003, *p* = 0.011), and time spent using a computer (beta = −8.89E‐05, se = 0.002, *p* = 0.962) (Table S3A).

We also found that the increase in blood free cholesterol is associated with an increased frequency of epileptic seizures (OR 1.001, 95% CI 1.00003–1.00214; *p* = 0.044; Figure 2). In this analysis, the Cochran *Q* test and *I* ^2^ were not significant (Cochran *Q*‐derived *p* = 0.71; *I* ^2 ^= 0). The horizontal pleiotropy analysis did not yield any positive results (*p* = 0.767; Table S5A).

In addition, drinking alcohol, sleep, physical activity, watching TV, steroid hormones, Gamma‐linolenic acid (18:3n6), and other potential factors showed no causal relationship with epilepsy, generalized convulsive epilepsy, or focal epilepsy (data not shown, Tables S3–S, 6).

### 3.2 Generalized convulsive epilepsy

**FIGURE 3:**
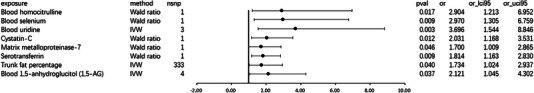
Odds ratios assessing the links between genetically predicted exposure and the occurrence of generalized convulsive epilepsy. The estimates correspond to Blood homocitrulline, Blood selenium, Blood uridine, Cystatin‐C, Matrix metalloproteinase‐7, Serotransferrin, Trunk fat percentage, and Blood 1,5‐anhydroglucitol (1,5‐AG).

The MR IVW analysis genetically indicated that blood uridine exhibited a suggestive causal relationship with generalized convulsive epilepsy (OR 3.696, 95% CI 1.544–8.846; *p* = 0.003; Figure 3). The Cochran *Q* test and *I* ^2^ did not suggest heterogeneity (Cochran *Q*‐derived *p* = 0.853; *I* ^2 ^= 0; Table S4B), and the MR‐Egger intercept excluded horizontal pleiotropy (*p* = 0.683; Table S5B).

Hippocampus volume appears to exhibit a suggestive causal relationship with generalized convulsive epilepsy (OR = 4.130, 95% CI 1.026–16.619; Table S3B). However, due to the limited number of two SNPs, subsequent sensitivity tests cannot be conducted, rendering the results unreliable. Amygdala volume (beta = −0.237, se = 1.310, and *p* = 0.86; Table S3B) does not demonstrate a suggestive causal relationship with generalized convulsive epilepsy.

### 3.3 Focal epilepsy

**FIGURE 4:**
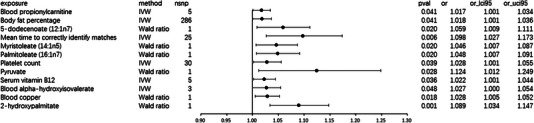
Odds ratios assessing the links between genetically predicted exposure and the occurrence of focal epilepsy. The estimates correspond to Blood propionylcarnitine, Body fat percentage, 5‐dodecenoate (12:1n7), mean time to correctly, identify matches, Myristoleate (14:1n5), Palmitoleate (16:1n7), Platelet count, Pyruvate, Serum vitamin B12, Blood alpha‐hydroxyisovalerate, and Blood copper, 2‐hydroxypalmitate. MR, Mendelian randomization; PheWAS, phenome‐wide association study; SNP, single‐nucleotide polymorphisms.

MR analysis suggested a suggestive association between blood propionylcarnitine (OR: 1.017, 95% CI 1.001–1.034; *p* = 0.041; Figure 4) and focal epilepsy, and the data indicated neither heterogeneity (Cochran *Q*‐derived *p* = 0.477; *I* ^2 ^= 0; Table S4C) nor horizontal pleiotropy (MR‐Egger intercept, *p* = 0.836; Table S5C).

The highly scrutinized factor of hippocampus volume (beta = −0.01, se = 0.031, and *p* = 0.75; Table S3C) does not show any indicative causal relationship with generalized convulsive epilepsy. The relationship between SNPs of amygdala volume and focal epilepsy was not included in the study due to not meeting the assumptions of MR. Blood decanoylcarnitine (beta = −0.009, se = 0.008, and *p* = 0.235; Table S3C) did not show a causal relationship in any of our three datasets, as we used a more stringent threshold and leave‐one‐out analysis also did not identify any SNPs that contributed to the causal relationship.

## 4 DISCUSSION

Epilepsy is one of the most prevalent brain disorders and carries a significant disease burden. Therefore, the identification of effective strategies to prevent epilepsy holds significant implications for enhancing public health at the population level. To the best of my knowledge, this is currently the most comprehensive MR study on the etiology of epilepsy. We included 316 phenotypes to provide a comprehensive etiological analysis of epilepsy. These phenotypes encompass highly relevant health‐related inquiries, such as environmental factors, lifestyle, blood, and imaging biomarkers.

In this study, the frequency of tiredness or lethargy in the last 2 weeks may be related to the occurrence of seizures, which is consistent with the complaints we received from patients in clinical practice (Kasteleijn‐Nolst Trenité, 2012). Fatigue is common among epilepsy patients (Kwon et al., 2017) and can occur both between seizures and after seizures (Hamelin et al., 2010). A study by Kasteleijn‐Nolst Trenité (2012) found that 53%−65% of adolescents and adults with epilepsy reported that their seizures were sometimes or entirely triggered by internal factors such as fatigue. Additionally, most studies indicate that 70%−90% of patients report at least two triggering factors (Nakken et al., 2005). This is not surprising, as there is a correlation between stress, sleep deprivation, and fatigue, making fatigue a part of mixed types of triggers (Ferrie et al., 1994; İşcan Ayyildiz & Bingöl, 2024; Frucht et al., 2000). However, different types of physical labor can have different effects on seizure outcomes, as heavy manual labor and prolonged computer use did not show an increased risk of epilepsy. Additionally, this result was not replicated in the other two types of epilepsy. From a genetic analysis perspective, there is no strong evidence to suggest that fatigue can induce epilepsy or that taking more rest can treat epilepsy in a general way.

To my knowledge, this is the first study to report a causal relationship between blood‐free cholesterol and the risk of epilepsy. Numerous studies have reported that hypercholesterolemia induces oxidative stress. High levels of free radicals exceeding the antioxidant defense mechanisms can cause irreversible damage to proteins, lipids, and DNA. Although previous research suggested that brain cholesterol metabolism is separate from the systemic circulation, during oxidative stress, plasma hydroxycholesterol (a hydroxylated metabolite of cholesterol) may have a detrimental impact on the blood–brain barrier (Dias et al., 2014). Additionally, high blood cholesterol can work in concert with hypertension to recruit leukocytes and platelets in the cerebral microcirculation (Rodrigues et al., 2014), inducing a pro‐inflammatory phenotype. Oxidative stress and neuroinflammation are critical pathological foundations for the occurrence of epilepsy (Łukawski & Czuczwar, 2023; Vezzani et al., 2019).

Consistent with previous MR research findings (Cai et al., 2022), we found a causal relationship between blood uridine and generalized convulsive epilepsy. In the human body, uridine is the precursor for the synthesis of uridine diphosphate (UDP). Reducing the synthesis of UDP can decrease neuronal excitability and sodium channel activity, thereby exerting an antiepileptic effect. This is precisely a part of the mechanism of action of lamotrigine in the treatment of epilepsy. There are also reports that genetic variations in CAD (a specific gene mutation, c.98T4G, p.Met33Arg) can lead to a disruption in uridine synthesis, which can also cause a special type of epilepsy and can be treated by supplementing with uridine (Koch et al., 2017; McGraw et al., 2021). The contradictory nature of uridine's properties is also evident in its effects on fatty liver (Le et al., 2014; Le et al., 2013). Our current MR studies cannot confirm the role of blood uridine in the risk of epilepsy in patients with CAD genetic variations. One major reason is that such patients are very rare, making it difficult to obtain GWAS data. It should be noted that the contradictory effects of blood uridine should not be ignored.

In a study involving patients with cblC deficient methylmalonic acidemia and hydrocephalus, characterized by high blood propionylcarnitine, it was observed that 61.8% of patients had epilepsy, and treatment with l‐carnitine resulted in symptom improvement (He et al., 2020). In our own MR study, we have also found a suggestive causal relationship between elevated blood propionylcarnitine levels and focal epilepsy. Previous MR studies have identified a causal association between decanoylcarnitine and epilepsy. In other MR studies, a causal relationship between decanoylcarnitine and epilepsy has been found (Cai et al., 2022). Although decanoylcarnitine was included in our study, we did not obtain a positive result. However, it is worth noting that we implemented stricter thresholds in our analysis. These findings suggest that different types of carnitine may have varying effects on epilepsy. The potential use of l‐carnitine as a treatment for epilepsy requires further exploration, and one possible mechanism of action could involve competitive inhibition.

Drinking alcohol, sleep, physical activity, watching TV, steroid hormones, Gamma‐linolenic acid (18:3n6) (Vaddadi, 1981), and other highly focused factors showed no causal relationship with epilepsy, generalized convulsive epilepsy, or focal epilepsy. Although hippocampal sclerosis, characterized by a reduction in hippocampal and amygdala volume, being the most common pathological basis for drug‐resistant mesial temporal lobe epilepsy (Malmgren & Thom, 2012; Reddy et al., 2019). Our data do not suggest a causal relationship. One possible explanation is that these two exposures are not the cause of epilepsy, but rather the outcome. Alternatively, they may impact the occurrence of epilepsy through other mechanisms.

However, this study has several limitations. Our study cannot include all exposures and SNPs. Further research is necessary to investigate additional factors that impact epilepsy and to obtain reliable results. Furthermore, there is controversy surrounding the establishment of thresholds, with no unified standard. Utilizing stricter thresholds may result in the exclusion of meaningful exposure factors. Instead, we aim to identify these potential risk factors through more profound and higher quality methods, rather than resorting to lowering the threshold. However, such an approach clearly necessitates long‐term and in‐depth research to achieve.

Notwithstanding these limitations, our utilization of MR serves as a means to examine whether genetic instruments independently corroborate potential protective associations between those 316 phenotypes and the risk of epilepsy. By employing a novel approach that utilizes genetic variants as IVs for causal inference, we are able to overcome the common challenges associated with observational research. This research outcome provides an initial response to the public's concern about preventive healthcare measures for epilepsy from the perspective of genetic analysis.

## 5 CONCLUSION

The study currently holds the most comprehensive coverage of influencing factors in epilepsy among MR studies. Our findings indicate that there was no statistically significant casual association between any of the 316 phenotypes and epilepsy, but the following factors have a suggestive causal risk for epilepsy: frequency of tiredness or lethargy in the last 2 weeks, blood free cholesterol, blood uridine, and blood propionylcarnitine.

## AUTHOR CONTRIBUTIONS

**Wei Zhang**: Writing—review and editing; resources; visualization. **Li‐ming Zhang**: Data curation; formal analysis; writing—review and editing. **Lin Zhi**: Writing—review and editing; investigation. **Ji Qi**: Writing—review and editing; formal analysis; supervision; validation. **Jue He**: Conceptualization; methodology; software; data curation; project administration; writing—original draft; writing—review and editing.

## CONFLICT OF INTEREST STATEMENT

The authors declare no conflicts of interest.

### FUNDING INFORMATION

This study did not receive any funding.

### PEER REVIEW

The peer review history for this article is available at https://publons.com/publon/10.1002/brb3.3602.

## Supporting information

Table_1A: r^2^ and F_static of Risk factors

Table_2A: Effect allele, frequency, effect on exposure and strength of association with epilepsy for SNPs

Table_3A: Causal estimates from Wald ratio, IVW‐RE, each exposure and Epilepsy risk.

Table_4A: The result of the heterogeneity of Epilepsy

Table_5A: Results of MR‐Egger analysis of potential bias in causal estimates of Epilepsy

Table_6C: Leave‐one‐out analysis results of Focal epilepsy

## Data Availability

Exposure data were obtained from the study by Charlie et al. (Saunders et al., 2021), which is a meta‐analysis of eight independent GWAS studies (UK [Cardis et al., 2007], French [Sanson et al., 2011], German [Kinnersley et al., 2015], MDA [Shete et al., 2009], UCSFSFAGS [Shete et al., 2009], GliomaScan [Rajaraman et al., 2012], GICC [Amirian et al., 2016], and UCSF/Mayo [Wrensch et al., 2009]) detailed in Table S1–S3. The outcome data that support the findings of this study are available in GWAS Catalog at: https://www.ebi.ac.uk/gwas/, [GCST90038645]; https://www.ebi.ac.uk/gwas/studies/GCST90038645, [GCST90043753]; https://www.ebi.ac.uk/gwas/studies/GCST90043753, [GCST007349]; and https://www.ebi.ac.uk/gwas/studies/GCST007349.
